# High-throughput screening to discover inhibitors of the CarD·RNA polymerase protein–protein interaction in *Mycobacterium tuberculosis*

**DOI:** 10.1038/s41598-020-78269-3

**Published:** 2020-12-04

**Authors:** Maxwell A. Stefan, Glory M. Velazquez, George A. Garcia

**Affiliations:** Department of Medicinal Chemistry, University of Michigan, Ann Arbor, MI USA

**Keywords:** Transcription factors, Drug discovery and development, Screening, Target identification

## Abstract

Multidrug-resistant *Mycobacterium tuberculosis* (MDR-TB) accounts for 3.7% of new cases of TB annually worldwide and is a major threat to global public health. Due to the prevalence of the MDR-TB and extensively drug resistant tuberculosis (XDR-TB) cases, there is an urgent need for new drugs with novel mechanisms of action. CarD, a global transcription regulator in MTB, binds RNAP and activates transcription by stabilizing the transcription initiation open-promoter complex (RPo). CarD is required for MTB viability and it has highly conserved homologues in many eubacteria. A fluorescence polarization (FP) assay which monitors the association of MTB RNAP, native rRNA promoter DNA and CarD has been developed. Overall, our objective is to identify and characterize small molecule inhibitors which block the CarD/RNAP interaction and to understand the mechanisms by which CarD interacts with the molecules. We expect that the development of a new and improved anti-TB compound with a novel mechanism of action will relieve the burden of resistance. This CarD FP assay is amenable to HTS and is an enabling tool for future novel therapeutic discovery.

## Introduction

The *Mycobacterium tuberculosis* RNA polymerase (MTB RNAP) is an attractive therapeutic target, as evidenced by the fact that inhibitors of RNAP are very effective bactericidals^1,2^. Several bacterial RNAP inhibitors have been identified over the last few decades; however, only two have been used clinically against MTB, Rifampin and Fidaxomicin. Unfortunately, resistance to these inhibitors has developed in *M. tuberculosis* (MTB)^3^. Traditionally, a majority of therapeutic interventions targeting RNAP have focused directly on enzymatic activity or by preventing RNAP interaction with DNA; however, RNAP functionality in vivo is significantly more complex, requiring several trans-acting factors which are essential for proper gene regulation and viability^4,5^.

**Figure 1:**
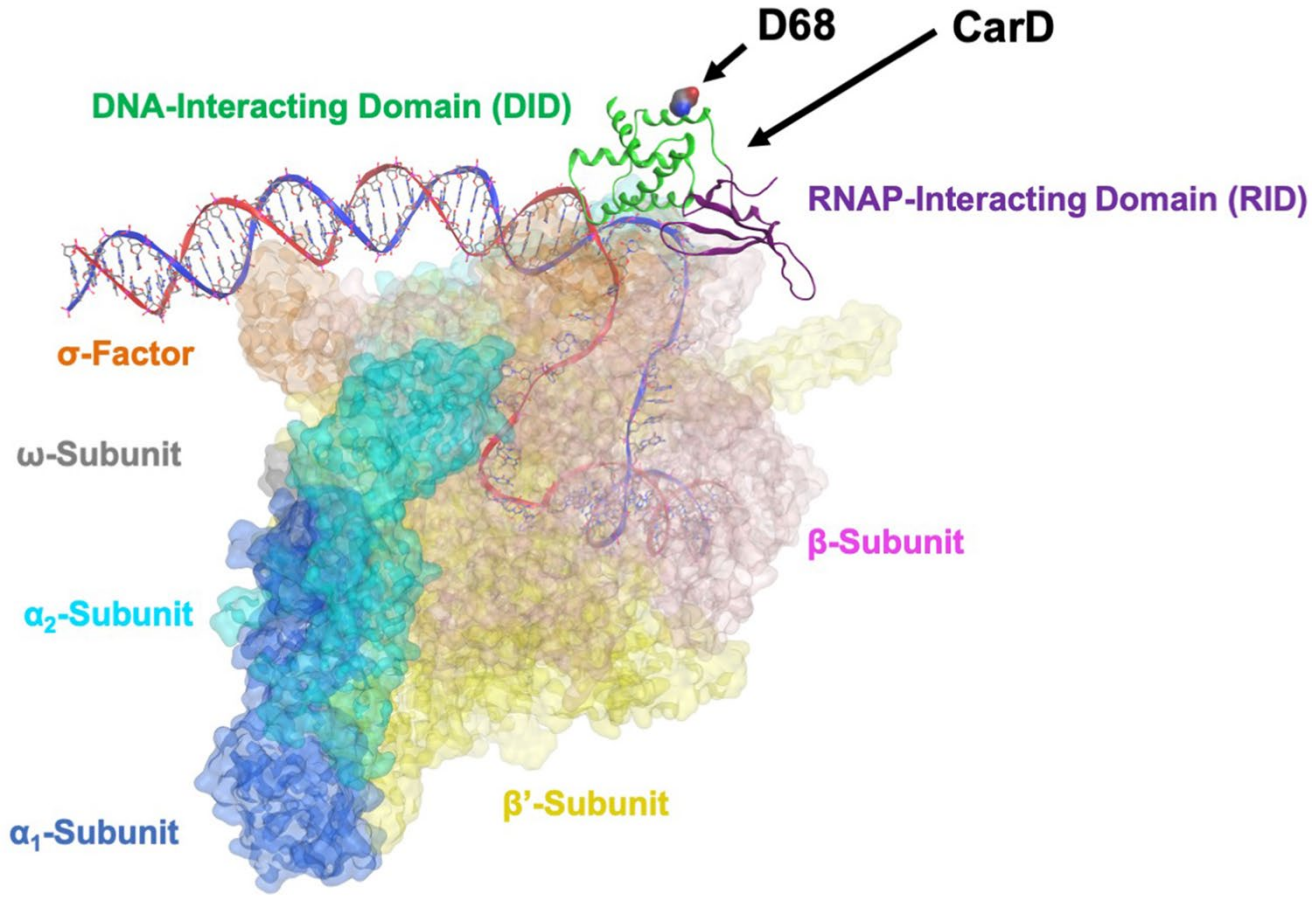
Structure of the MTB CarD Complex with RNAP·DNA Open Promoter Complex (RPo) and RbpA. The RNAP, RbpA and CarD subunits are labeled. The position of CarD D68 is indicated by the arrow. This structure is from PDB: 6EDT (Darst, S.A., Campbell, E.A., Boyaci Selcuk, H., and Chen, J., https://doi.org/10.2210/pdb6EDT/pdb).

In MTB, CarD is a global regulator that modulates transcription by stabilizing the RNAP open promoter complex (RPo)^6,7^. CarD consists of two subdomains, an N-terminal domain (1–53) which interacts with MTB RNAP at the β1β2-lobes of the β-subunit, also known as the “protrusion” and a C-terminal domain (64–162), which is separated from the N-terminal domain by a 10-amino acid linker. The α-helical C-terminal domain has been shown to interact with promoter DNA at the upstream fork junction (Fig. 1)^8,9^. CarD’s role is more complex than that of a monotonic transcriptional activator. It has been shown that CarD can activate *and* repress transcription from different promoters^10^. We^11^ and others have found that activation occurs when CarD stabilizes the RPo of promoters that have inherently short RPo lifetimes to facilitate transcription initiation. Whereas it has been suggested that CarD stabilization of promoters with inherently stable RPo inhibits promoter escape and represses the expression of genes. From these studies, it was determined that about two-thirds of the MTB genome are differentially expressed if CarD activity is altered, suggesting a critical role for CarD in MTB homeostasis^10^. CarD is required for MTB viability and is involved in mediating stress responses such as exposure to antibiotics and oxidative stress^4,12^. CarD’s function as a global transcriptional regulator that is required for MTB survival makes it an attractive and novel potential therapeutic target.

Herein we discuss the development, optimization, and the validation of a fluorescence polarization assay to monitor the interaction between CarD and the RNAP. A high throughput screen (HTS) comprising 23,320 small molecules was performed. Hits from this screen were characterized in both biochemical and biophysical assays for validation and to probe their mechanism(s) of action. This screen and secondary assays represent a robust method for identifying inhibitors for the interaction between CarD and the RNAP as well as DNA binding to RNAP.

## Results

### CarD fluorescence polarization assay

**Figure 2:**
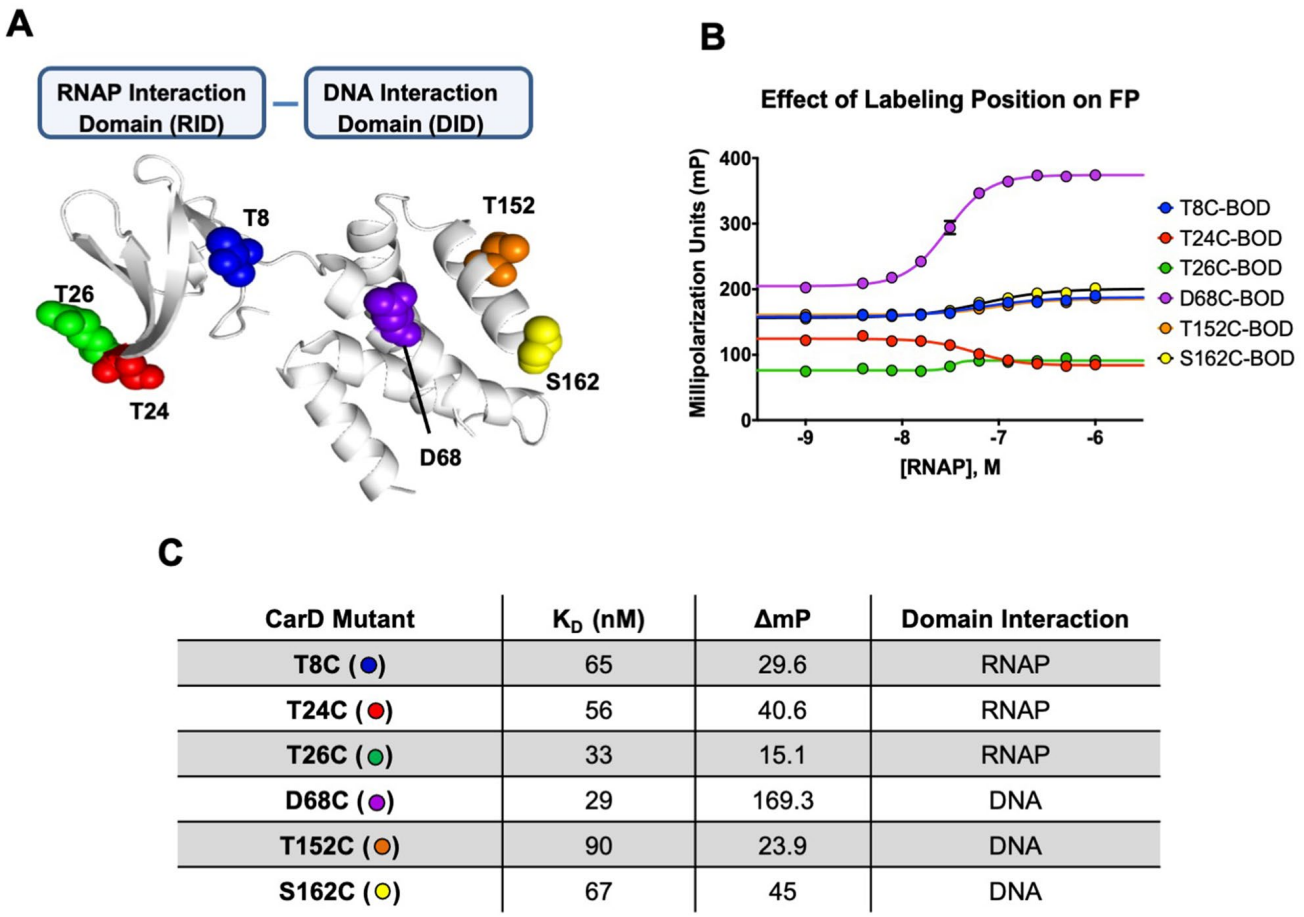
Characterization of labeling sites on CarD. (**A**) Sites on CarD which were selected for site-specific mutagenesis and subsequent labeling with BODIPY-FL (colors correspond with binding isotherms in (**B**). (**B**) Binding isotherms for CarD mutants labeled with BODIPY-FL (note: several differences in this assay are as follows: 50 mM KGlu. 0.001% Triton X-100, and 12.5 nM CarD-BODIPY-FL were used in this experiment). (**C**) Summary of isotherm parameters for each CarD variant as well as domain interaction on the RPo where the BODIPY-FL probe is located.

To develop the fluorescence polarization (FP) assay, site-specific mutagenesis of several selected residues on CarD to cysteine was performed followed by chemical modification by BODIPY FL iodoacetamide and DAOTA haloacetate. Labeling efficiency of the CarD mutants varied from ~ 40 to 100% (Supplemental Table S2). Six labeling sites on both the N-terminal RNAP-interacting domain (RID) and the C-terminal DNA-interacting domain (DID) were explored. There were several criteria for the selection of the labeling sites. The first and most important was to utilize existing structural data to avoid interfaces critical to the interaction between CarD and both RNAP and DNA. The second was to select a distribution of sites on CarD encompassing both domains distal to and near the inter-domain linker. Thirdly, was to preferentially select existing serines or threonines which were not adjacent to acidic amino acids (which can increase the pKa of the introduced Cys lowering labeling efficiency; conversely, proximity to a basic residue would help to drive the labeling reaction to completion). Substitution of a Ser/Thr with Cys would be considered conservative and likely to minimally impact CarD folding. Lastly, if the local environment were not ideal for Ser/Thr mutagenesis, solvent exposed residues adjacent to basic residues were selected (e.g., D68). The dynamic range and K_D_ of each of the BODIPY FL-labeled CarD mutants were characterized by titration with WT MTB RNAP (Fig. 2).

Several mutant labeled CarD’s produced discernable binding curves, although the FP dynamic range and affinity varied (11- and 3-fold, respectively) across the mutants (Fig. 2B,C). Of the six variants explored, labeling at position D68 produced a binding curve with the greatest dynamic range (ΔmP = 169.3) and had the strongest affinity for the RPo (K_D_ = 29 nM) under the conditions tested. Several other labeled CarD mutants also produced binding curves (T8C and S162C) though the dynamic ranges were significantly smaller (ΔmP ~ 40) and they had lower affinities (K_D_ ~ 65 nM), which may suggest the probe at those positions could be slightly perturbing CarD binding to RNAP·DNA. There were small responses, ΔmP = 23.9 and ΔmP = 15.1, for CarD T152C and CarD T26C respectively. For CarD T26C an inverse binding curve was observed, possibly due to the propeller effect on the BODIPY-FL probe upon binding to the RPo complex. Due to its highest affinity and largest FP dynamic range, the CarD D68C mutant was used for all further CarD FP studies.

Optimal binding conditions for the CarD·RPo interaction were explored as were the stability of the FP in the presence of DMSO and over time (Supplemental Fig. S10,S11**)**. Binding curves for CarD·RPo were determined in titration experiments with both NaCl and potassium glutamate (KGlu). Increasing concentrations of NaCl resulted in complex destabilization above 100 mM. The ternary complex was significantly less sensitive to KGlu with clearly defined curve contours up to 500 mM KGlu, although a decrease in dynamic range (~ 30% for 500 vs 50 mM) is evident. Final salt conditions were 150 mM KGlu and 10 mM MgCl_2_. Addition of DMSO to ternary complex at RNAP concentrations (1.25 × the K_D_ or 15 nM, below) shows that the assay can tolerate DMSO up to 5% without significant deviation from the negative control (Supplemental Fig. S10). The assay signal was also found to be stable over 24-h (Supplemental Fig. S11).

**Figure 3:**
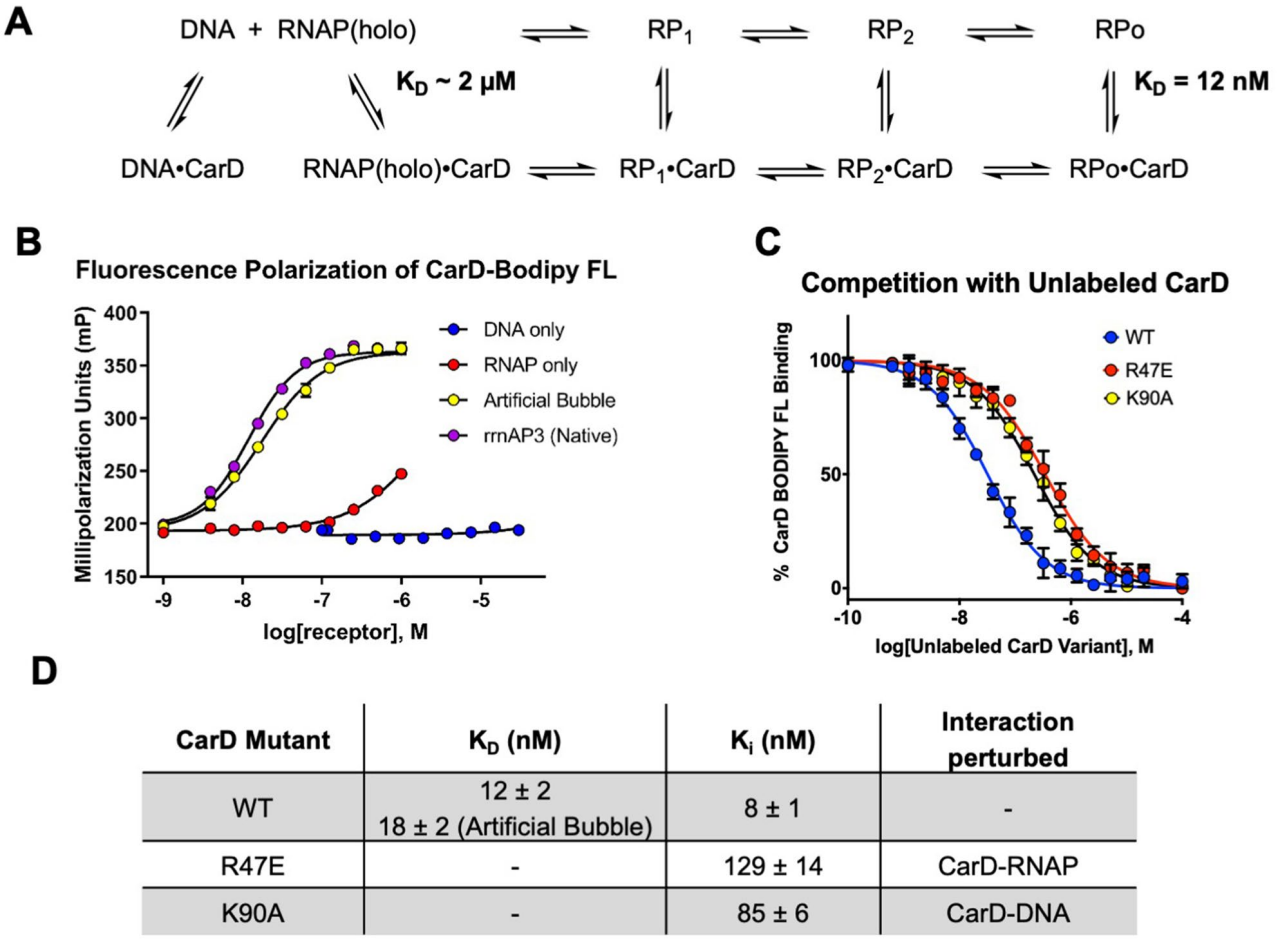
Characterization and validation of CarD Fluorescence Polarization Assay. (**A**) Schematic of CarD binding partners with dissociation constants derived from (**B**). (**B**) FP of CarD-BODIPY-FL (3 nM) with RNAP and DNA. CarD shows essentially no binding to DNA at DNA concentrations up to 30 µM (data not shown). (**C**) Competition curve showing displacement of CarD-BODIPY-FL by WT and mutant CarD proteins (RNAP present at 1.25 × K_D_ as determined in (**B**). (**D**) Summary of binding and inhibition constants as determined using Eq. (5).

Binding curves were produced using labeled CarD with promoter DNA (61 base pair rrnAP3 fragment) only, RNAP only, and RNAP with both native rrnAP3 DNA and a mutant of the rrnAP3 DNA with an artificial bubble from -11 to + 1 (Supplemental Table S1, Fig. 3B). Dissociation constants (K_D_) for each of the components mentioned above were determined (Fig. 3A,B). No polarization response was observed for promoter DNA only up to 30 µM (Fig. 3B). RNAP only (no DNA) concentrations were tested up to 1 µM; however, a complete binding curve could not be generated from the concentrations tested (Fig. 3B). An estimate of ~ 2 µM for the RNAP·CarD K_D_ was made by extrapolating the RNAP only binding curve to the maximum mP observed in the presence of DNA (Fig. 3B). The affinity for the RPo using the “native” rrnAP3 DNA fragment was determined to be 12 ± 2 nM and for the artificial bubble DNA 18 ± 2 nM (Fig. 3D).

### Competition and preliminary inhibition experiments

Competition experiments were performed using 15 nM RNAP (1.25 × K_D_) and rrnAP3 DNA template at 300 nM which was determined to be the minimal amount of DNA needed to maintain RPo complex stability (Supplemental Fig. S12). The inhibition constant (K_i_) for WT unlabeled CarD was determined to be 8 ± 1 nM, which is similar to the K_D_ of CarD-BODIPY-FL for the “native” RPo complex, indicating that the BODIPY probe does not interfere with CarD binding to RPo (Fig. 3C,D). Two mutations were studied as controls because they were shown in vivo to negatively impact MTB viability or function of CarD in MTB^13,14^. Inhibition constants were determined for both CarD R47E, which disrupts the CarD N-terminal interaction with RNAP, and CarD K90A, which has been shown to disrupt CarD interactions with promoter DNA. Consistent with those reports, CarD R47E (K_i_ = 129 ± 14 nM) and CarD K90A (K_i_ = 85 ± 6 nM) show decreased affinity for the RPo complex (Fig. 3C,D). These results indicate that there is a narrow window in which CarD can be disrupted before you start to negatively impact MTB homeostasis/viability.

**Figure 4:**
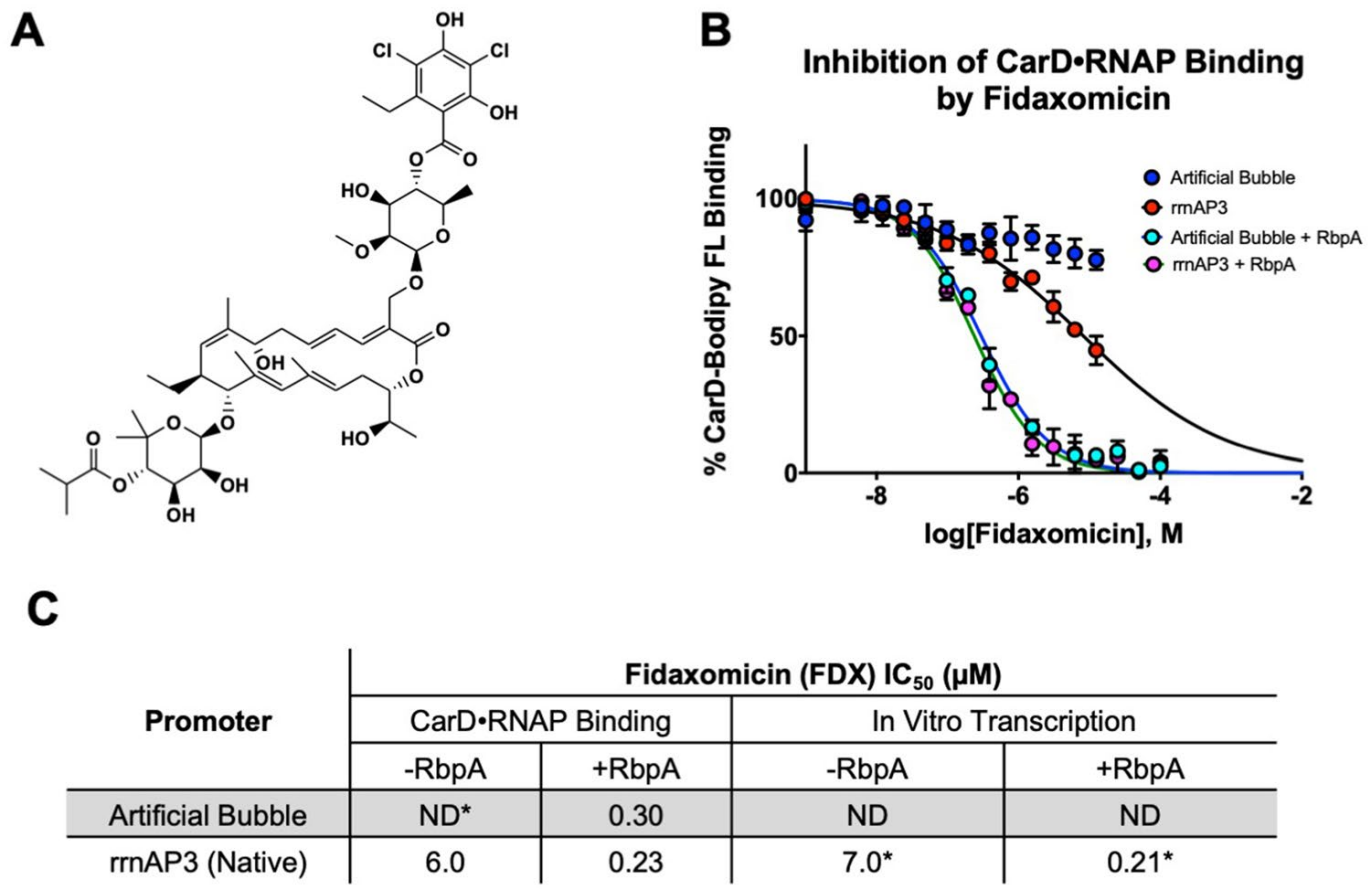
Fidaxomicin inhibition of CarD association with RPo. (**A**) Structure of Fidaxomicin. (**B**) CarD binding to RPo in the presence of varying concentration of FDX with “native” rrnAP3 and artificial bubble DNA. (**C**) Summary of inhibition data with and without RNA polymerase binding protein with both the CarD FP compared to literature values.

To test the capability of the CarD FP assay to monitor the presence of DNA within RNAP, the IC_50_ of Fidaxomicin was determined (Fig. 4). Fidaxomicin (FDX) binds to the “switch” domain of RNAP locking it in an “open” clamp conformation preventing DNA from being securely bound within RNAP^15–17^. This experiment was conducted with both “native” and artificial bubble DNAs as well as in the presence and absence of *M. smegmatis* RNA polymerase binding protein, RbpA, which is required for Fidaxomicin potency against mycobacterial RNAPs. In the absence of RbpA, the observed IC_50_ is 6 µM which is in agreement with the 7 µM inhibition observed with FDX in an in vitro transcription assay^16^. Interestingly, the IC_50_ determined with the artificial bubble DNA is significantly higher (IC_50_ > 80 µM), the artificial bubble template shifts the equilibrium of RPo toward the “closed” clamp configuration which has a dramatically lower affinity for FDX. In the presence of RbpA, the “native” DNA template exhibits an increase in FDX potency to an IC_50_ of 0.23 µM, which is consistent with other results recently reported^15^. In the presence of RbpA and the artificial bubble template, the IC_50_ for FDX is 0.30 µM, which indicates that RbpA is able to overcome the reduction in affinity for FDX observed when using the artificial bubble template (Fig. 4).

### Primary high throughput screen

**Figure 5:**
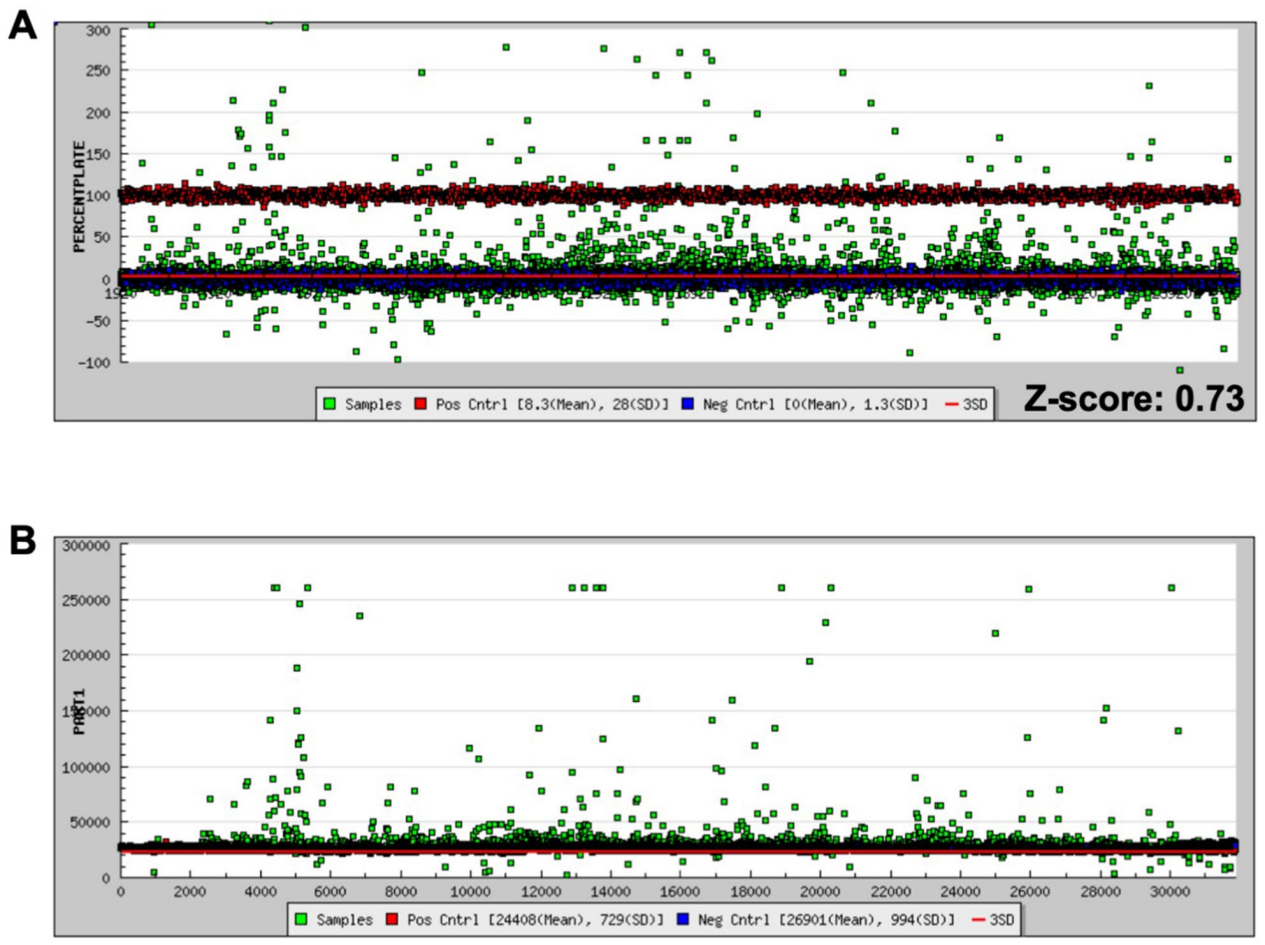
HTS of Maybridge 24 K compound library with the CarD-BODIPY-FL FP RNAP binding assay. (**A**) Percent activity and (**B**) parallel intensity of primary screen results (Positive controls (Red), negative controls (blue), and samples (green)).

A 23,320-compound small molecule library (Maybridge 24 K) was screened using the CarD-BODIPY-FL FP assay (Fig. 5A). For the primary screen 10 nM WT MTB RNAP (1.33 × K_D_) was used which resulted in a dynamic range of ΔmP = 76.9. The Z-score for the primary screen was 0.73. Several criteria were used to identify hits for this screen (see progression flowchart in Supplemental Fig. S13), 995 compounds or 4.26% were at least 3 standard deviations (3σ) from the negative controls; however, there were a significant fraction of these which were found to be from compound interference with the assay (Fig. 5B) and others which were red and black flagged by NIH filters. Compounds which were > 110% percent active (relative to the positive control on a plate-by-plate basis) were eliminated from the hit pool (96 compounds removed). These compounds are either false positives from fluorescence interference or may be causing denaturation of the proteins in the assay. Additionally, compounds were eliminated from the hit pool if they caused an increase in the parallel intensity > 3σ from the average of the positive and negative controls or 29,600 RFU (357 compounds removed). Compounds which were designated as NIH red or black flag compounds were eliminated from the final count (62 compounds removed). Finally, a promiscuity filter was applied to eliminate compounds which hit in more than 9 other screens previously conducted at the screening facility (58 compounds removed). After all filters were applied the final hit rate was 1.8% (422 / 23,320), which is substantially higher than the anticipated 1% hit rate.

### Confirmation, counter screen, and concentration response screen

**Figure 6:**
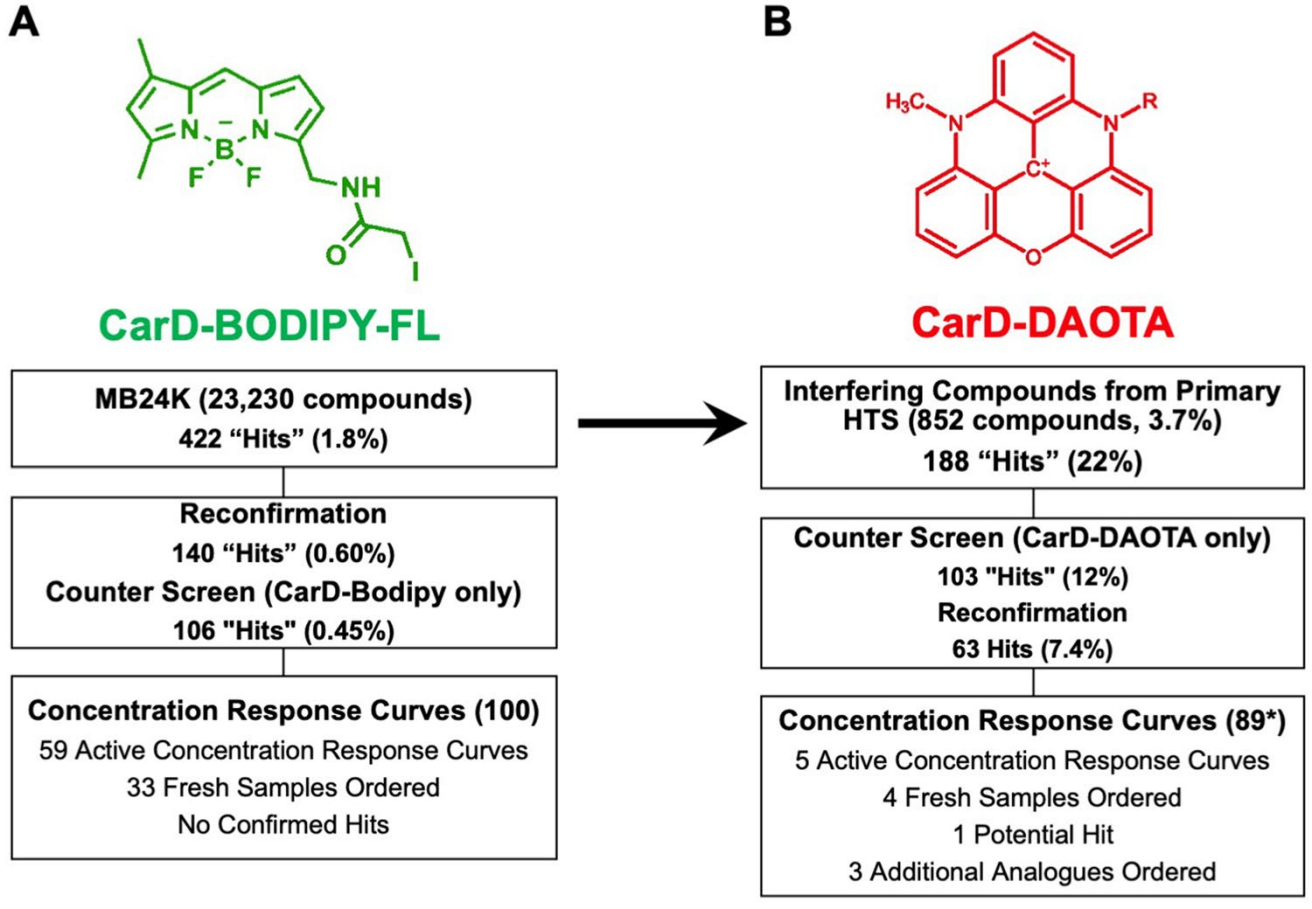
CarD-BODIPY-FL and CarD-DAOTA screening summary. **A)** BODIPY-FL Structure, CarD-BODIPY-FL Triage Chart. **B)** DAOTA Structure, CarD-DAOTA Triage Chart.

All 422 compounds which were designated “hits” from the primary screen were re-tested in triplicate from the same stock plates. The Z-score for the retest plates was 0.76 (Supplemental Fig. S14). Compounds which were > 3σ from the negative controls in triplicate were considered confirmed hits (202 compounds removed). Additionally, compounds which were greater than or equal to 3σ parallel intensity of the negative control and equal or less than 3σ of the positive control were advanced (30 and 11 compounds removed respectively). All flagged PAINs (pan-assay interference compounds) and compounds with a clogP > 5 were removed (39 compounds removed total). At this point 140 compounds remained (0.60% of the primary screen). A counter screen was conducted to eliminate compounds which interfered with CarD-BODIPY-FL parallel fluorescence intensity (24 compounds removed) and fluorescence polarization (10 compounds removed) by more than 3σ. At this stage there were 106 compounds, or 0.45% of the primary screen remaining. Of the 106 compounds 100 were selected for concentration–response studies (CRC), 59 compounds were designated as a positive result in the CRC study (Fig. 6A and Supplemental Fig. S13) and, of these 33 fresh samples were ordered.

### Reconfirmation

All 33 fresh powder samples were characterized by LCMS. Two of the 33 compounds were found to be either completely degraded or their MW did not match the labeled compound. The other 31 fresh powders did match the MWs of the labeled compounds; however, the purities ranged from < 50% to > 95%, with the vast majority being the latter. Of the 31 compounds tested, 11 did not reconfirm and several other compounds had extremely weak inhibition (> > 200 µM). Eight compounds were initially considered as hits either because they had an IC_50_ below 200 µM or they were part of a structural cluster (Supplemental Table S3). The challenge of compound aggregation producing false positives during HTS campaigns has been demonstrated by Shoichet and co-workers^18^. We routinely examine the Hill slopes of our inhibition fits because false positives due to aggregation tend to have abnormally high Hill slopes. The Hill Slopes for the 8 compounds were >  > 1 and subsequent solubility curves via turbidity analyses matched the inhibition curves indicating that these were indeed false positives due to aggregation.

### DAOTA-CarD screen

**Figure 7:**
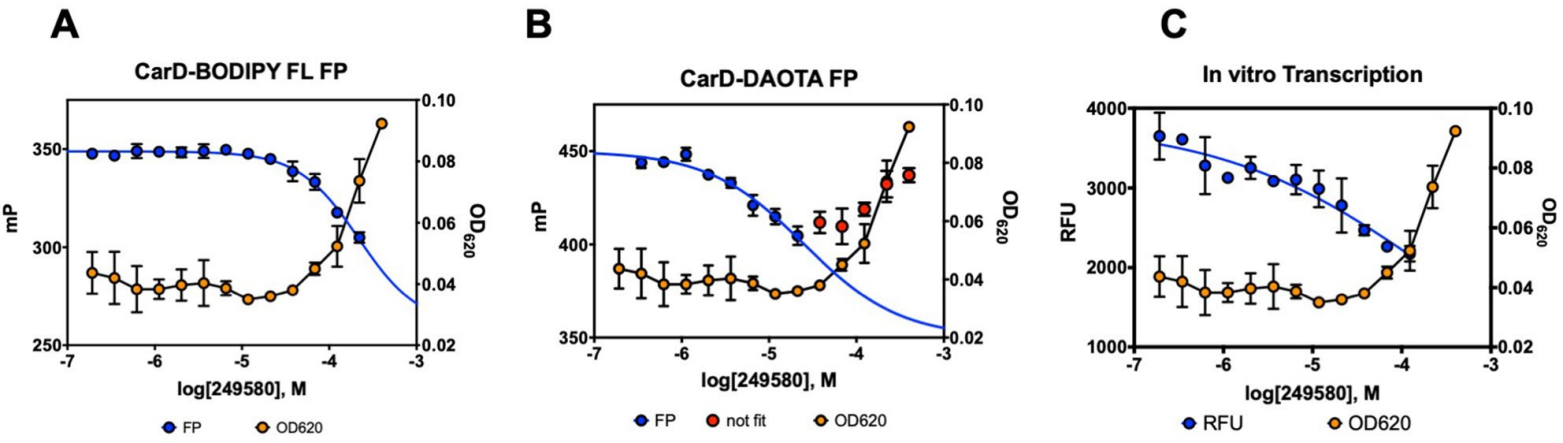
CCG-249580 activity in CarD FP and in vitro transcription assays and binding model. (**A**) CarD-BODIPY-FL activity and turbidity plots. (**B**) CarD-DAOTA activity and turbidity plots. (**C**) Inhibition of in vitro transcription by CCG-249580. *mP* milli polarization units, *RFU* relative fluorescence units.

Compounds that exhibited fluorescence interference with the green probe, as evidenced by very high parallel fluorescence intensity (Fig. 5B), were tested again using CarD labeled with a red-shifted fluorophore (DAOTA, Fig. 6B). For hit compound identification, we implemented the criteria of the primary screen to this secondary HTS campaign. A total of 852 molecules were tested with DAOTA-labeled CarD, from which 188 compounds showed no interference with the red probe. CRC experiments were conducted with 89 hit compounds identified from the reconfirmation study. Five compounds exhibited IC_50_ < 200 µM. A reconfirmation with fresh powder compounds was performed as described above, and only one compound (CCG-249580, Supplemental Table S4) presented activity in both CarD FP assays (Table 1 and Fig. 7**)**. Three analogs of CCG-249580 were ordered and tested with the CarD FP assays, however, no significant activity was obtained from these compounds (Table 1).

**Table 1:** Two hits from the CarD-DAOTA screen and three CCG-249580 analogues.

Name	Structure	CarD-BODIPY-FL assay		CarD-DAOTA assay	
		CCG sample IC_50_ (µM)	Powder sample IC_50_ (µM)	CCG sample IC_50_ (µM)	Powder sample IC_50_ (µM)
CCG-237488		25	ND	20	ND
CCG-249580		263	202	32	45
019–738-824		N/A	> 100*	N/A	> 100
008–371-831		N/A	> 100	N/A	> 100
046–418-303 (Ridinilazole)		N/A	> 100	N/A	> 100

N/A: not applicable, compounds were not in CCG library. N/D: not determined.

*Compounds were inactive up to 100 µM. They exhibited inhibition above 100 µM which is likely artifactual due to compound aggregation.

### In vitro transcription

The in vitro transcriptional activity of the RNAP in the presence of CCG-249580 was determined by performing the MGA transcription assay (Fig. 7). While the data clearly show an increasing inhibition trend with increasing compound concentration, they are insufficient to determine an IC_50_; however, there is significant inhibition at much lower concentrations than those that show inhibition of CarD binding. When we compare the OD_620_ data (turbidity test) of CCG-249580 with the transcriptional activity data, it is clear that there is significant inhibition at concentrations that exhibit no or little turbidity (e.g., precipitation). Considering that CarD is not present in the reaction, this indicates that the compound is interacting directly with either MTB RNAP or DNA.

## Discussion

Identification of new antibiotics which act via novel mechanisms of action is critical for addressing rising levels of antibiotic resistance. While directly targeting RNA polymerase has been proven effective, transcription is a complex process which is highly regulated. We believe that this regulation represents an attractive target for novel antibiotic discovery. The transcriptional regulator, CarD is required for MTB viability^12,13,19^. While the effect of CarD on viability in other organisms has not been explored, its presence in other clinically relevant organisms suggests a unique opportunity to be a novel broad-spectrum target.

A highly robust fluorescence polarization assay which probes the interaction between CarD and the RPo (open-promoter complex) was developed and utilized to identify novel antibiotic chemical matter. CarD lacks endogenous cysteine residues which allows for site-specific incorporation of fluorescent probes using site-directed mutagenesis and thiol reactive conjugated probe (Fig. 2). Labeling efficiencies were > 90% in all cases with the exception of the CarD S162C mutant, where labeling was determined to be 40% by UV/Vis methods and 50% by LCMS (Supplemental Table S2). This is likely because cysteine at this position has a pKa which is significantly higher than the standard pKa ~ 8.0 of cysteine due to the proximity to the C-terminus. In preparing for the use of this assay for HTS, we found that CarD requires DNA to be present for high affinity interaction RNAP (12 nM vs 2 µM without DNA at 37 °C, Fig. 3). This binding affinity is consistent with the binding affinity predicted from rate constants determined using stop flow experiments^7^. Their reported value is 6.7 nM at 25 °C which is comparable to the dissociation constant obtained from our FP assay, 7.5 nM at 25 °C (Supplemental Fig. S11). The interaction of CarD with the RPo is roughly 170-fold tighter than in the absence of DNA. Endogenous concentrations of CarD in *M. smegmatis* are reported to be 1.6 µM as determined from quantitative immunoblotting, which suggests that CarD is likely partially bound to free RNAP in vivo.

Inhibition constants were determined to validate the FP assay and to assess whether the BODIPY probe interferes with the CarD RPo interaction. Competition of CarD-BODIPY-FL with WT unlabeled CarD resulted in a K_i_ = 8 nM, which is similar to the K_D_ determined for CarD-BODIPY-FL (Fig. 3). This suggests that the BODIPY probe has a negligible effect on CarD binding to the RPo. Competition experiments with two unlabeled CarD variants, R47E (which is at the interface between CarD and RNAP) and K90A (which has been shown in EMSA experiments to be involved in DNA binding) reveal that the interactions between CarD and both RNAP and DNA are compromised with these mutations respectively. This indicates that disruption of either of these interactions between CarD and the RNAP·DNA complex will reduce the affinity of CarD and thus destabilize the RPo complex.

The differential binding affinity of CarD for free RNAP (~ 2 µM) compared to its affinity for the RPo (12 nM) broadens the repertoire of types of inhibitors that can be identified with this assay (Fig. 3). We conducted the HTS at 10 nM labeled CarD, where there is negligible polarization response from free RNAP; therefore we should be able to identify inhibitors of DNA association with RNAP as well as inhibitors of CarD binding. This was shown to be the case with Fidaxomicin (FDX). FDX blocks the RNAP clamp closure onto DNA preventing the unwinding of DNA^15,17^. Recent reports demonstrated the involvement of another transcriptional regulator, RNA polymerase binding protein (RbpA), in high affinity inhibition of RNAP (IC_50_ = 0.21 µM) by FDX^15^. RbpA acts synergistically with FDX through interaction of RpbA’s N-terminal tail (NTT) with FDX. Using *M. smegmatis* RbpA (which shares > 90% sequence similarity to the MTB homolog) we found that we can monitor the effect of FDX on DNA binding in our CarD FP assay (IC_50_ = 0.24 µM) (Fig. 4). This expands the utility of this assay in drug discovery related to inhibition of transcription in MTB by finding not only inhibitors which target CarD, but also those which affect RNAP loading onto DNA.

A 23,320-compound library (MB24K) was screened using this CarD FP assay. The z-score was 0.73, demonstrating that the assay is highly suitable for HTS (Fig. 5A). From our screening campaign, only CCG-249580 was identified to have some inhibitory activity in reconfirmation assays (Fig. 7). However, the turbidity curve indicates that, at concentrations higher than 34 µM, CCG-249580 is prone to aggregation. Considering that the aggregation could cause a false positive^18^, we attempted to confirm a direct binding interaction between CarD and CCG-249580 by performing ^1^H–^15^N HMQC 800 MHz NMR experiments on ^15^N-labeled CarD. While we were able to get sufficiently resolved spectra, we did not observe any significant resonance shifts to support direct binding of CCG-249580 to CarD (Supplemental Fig. S18). However, the low potency of the compound (~ 100 µM) would result in a low % of CarD bound to compound at the solubility-limited concentration we could test, making it difficult to detect in the NMR experiment. CCG-249580 could cause inhibition in our FP assay by interfering with DNA loading, similar to FDX. We therefore tested CCG-249580 in our in vitro transcription assay^20^. Those results showed that CCG-249580 displayed concentration-dependent inhibition of transcription, which suggests that this compound is interacting directly with MTB RNAP or DNA (Fig. 7). It is possible that CCG-249580 may be interacting with and stabilizing the closed-promoter complex (RPc), indirectly interfering with the binding of CarD to the RPo. We purchased three analogs of CCG-249580 for further testing, (Table 1 and Supplemental Table S4). However, none of these showed any significant activity in either CarD FP assay (Table 1).

The bacterial RNAP is an attractive but complex antibiotic target. Most of the therapeutic interventions have been focused on targeting the enzymatic activity directly or preventing the RNAP interaction with DNA^21^. Targeting transcription has been largely effective for killing MTB, as indicated by the success of both Rifampin and Fidaxomicin, but few investigations have been centered on targeting CarD activity. CarD represents a new class of RNAP-binding protein that is present in mycobacteria and conserved in many other eubacteria. The approach described in this work can be used to identify and characterize small-molecule inhibitors with the capability of inhibiting the CarD/RNAP interaction, both directly and indirectly. Targeting transcription factors, such as CarD, may represent a new approach that can compromise pathogen viability by dysregulation of RPo complexes. CarD homologs are present in many bacterial phyla, such as *Chlamydiae* (*chlamydia*), *Firmicutes* (*C. diff. colitis*), and *Spirochaetes* (*Lyme disease*), making it potentially broad therapeutic target^10^. In MTB, CarD has been identified as an essential protein that has a role in the stringent response to oxidative stresses and starvation^4,12^. Considering the impact of CarD critical biological functions during tuberculosis*.* infection, CarD represents a critical target for treatment against tuberculosis and potentially other clinically relevant pathogens.

## Materials and methods

### Protein manipulation, purification, and labeling

Wild-type MTB CarD and RNAP were prepared as previously described^22^. Mutant CarD variants were produced by 2-step mutagenesis using primers described in Supplemental Table S1. For fluorophore-labeled CarD, BL21(DE3) *E. coli* transformed with pET19bbps-CarD plasmid was grown to an OD_600_ of 0.6 in 2xTY and induced with 1 mM IPTG for 4 h at 28 °C. Cells were harvested and pellets from 1L of culture were resuspended in 20 mL of lysis buffer (20 mM Tris HCl (pH 8.0), 500 mM NaCl, 0.1 mM EDTA, 5% glycerol (v/v), 5 mM β-mercaptoethanol (βME)) supplemented with 2 mM PMSF, 1X Roche Protease cocktail, 1 mg/mL lysozyme, and 200 U DNase I. Cells were disrupted by sonication and collected by centrifugation at 30,000×*g* for 45 min at 4 °C. Clarified lysate was sterile filtered and applied to a 1 mL HisTrap HP column. CarD was eluted by stepwise gradient to 500 mM imidazole after extensive washing.

CarD was precipitated with 0.3 g/mL ammonium sulfate followed by rinsing the protein pellet with labeling buffer (100 mM Tris HCl (pH 7.5 at 25 °C), 100 mM NaCl, and 1 mM EDTA) with the same concentration of ammonium sulfate. Precipitated CarD was resuspended in labeling buffer with a tenfold molar excess TCEP and allowed to incubate at 25 °C for 1 h. A tenfold molar excess of BODIPY-FL iodoacetamide (*N*-(4,4-difluoro-5,7-dimethyl-4-bora-3a,4a-diaza-s-indacene-3-yl)methyl)iodoacetamide, Thermo Fisher Scientific) or KU650 haloacetate (DAOTA, KU Dyes) suspended in dry DMF was added dropwise to the CarD solution with stirring. The reaction was allowed to proceed for 2.5 h before quenching with excess βME. Labeled CarD was further purified by application to 1 mL of NTA-Ni^2+^ agarose. The applied solution was eluted with 5 mL of lysis buffer with 500 mM imidazole after washing. *Precission* protease was added and the mixture was dialyzed overnight in SEC Buffer (20 mM Tris HCl (pH 8.0), 300 mM NaCl, 0.1 mM EDTA, 5% glycerol, and 2 mM DTT).

*Precission* protease was removed from the incubation mixture with 1 mL of glutathione agarose before being applied to a Superdex 200 10/300 GL column equilibrated with SEC buffer. The resulting labeled-CarD was stored at -80 °C. The degree of labeling was confirmed both spectroscopically using Eq. (1), where A_504_ is the absorbance of sample at 504 nm and ɛ_504_ is the extinction coefficient for BODIPY-FL (68,000 M^−1^·cm^−1^) as well as by LCMS (Supplemental Table S2 and Supplemental Fig. S1–S9). For DAOTA, the absorbance maximum is 560 nm and the extinction coefficient is 16,800 M^−1^ cm^−1^. Labeling efficiencies ranged from 40–100% and are described in Supplemental Table S2. Please refer to Supplemental Materials and Methods for protein purification of *M. smegmatis* RbpA.

1 $$ \frac{{{\text{A}}_{{{\text{dye}}}} }}{{{\upvarepsilon }_{{{\text{dye}}}} }}{ } \times { }\frac{{{\text{MW }}\;{\text{of }}\;{\text{CarD}}}}{{{\text{CarD }}\;{\text{mg}}/{\text{mL}}}} = { }\frac{{{\text{moles}}\;{\text{of}}\,{\text{ dye}}}}{{{\text{moles }}\;{\text{of }}\;{\text{CarD}}}} $$

### CarD fluorescence polarization assay

The fluorescence polarization (FP) assay was conducted as described below in a final volume of 20 µL. Optimized CarD FP assay buffer contains 20 mM Tris HCl (pH 7.9 at 37 °C), 150 mM potassium glutamate (KGlu), 10 mM MgCl_2_, 0.1 mM EDTA, 0.01% Triton X-100, 5 mM DTT unless noted otherwise. For binding curves used to determine dissociation constants (K_D_), BODIPY-FL-labeled CarD (CarD-BODIPY-FL) was tested at three fixed concentrations of 1 nM, 3 nM and 5 nM with 1 µM rrnAP3 and artificial bubble DNA fragments (the latter of which has a 12 base noncomplementary region from -11 to + 1 of the transcription start site (TSS), Supplemental Table S1). WT MTB RNAP was typically titrated from 1 µM to 244 pM. Assay components were allowed to incubate in a humidified incubator at 37 °C for 2 h before being read in a BioTek Synergy H1 Hybrid Multi-mode plate reader (excitation: 485/20 nm, emission: 528/20 nm). Fluorescence polarization (P) was determined by subtracting a blank well containing all assay components except CarD-BODIPY-FL from both the parallel (F_II_) and perpendicular (F_⊥_) intensities and Eq. (2) was used to determine polarization. FP values for binding curves were fit to a four-parameter sigmoidal dose–response with top and bottom limits unconstrained for each curve as described in Eq. (3), where Y = millipolarization (mP) and X = concentration of RNAP·DNA. K_D_ was determined by averaging the K_D_ determined at the 3 concentrations of CarD-BODIPY-FL, each conducted in triplicate.

2 $$ {\text{P}} = \frac{{{\text{F}}_{{{\text{II}}}} - {\text{F}}_{ \bot } }}{{{\text{F}}_{{{\text{II}}}} + {\text{F}}_{ \bot } }} $$

3 $$ {\text{Y = Bottom}} + \frac{{{\text{Top}} - {\text{Bottom}}}}{{1 + 10^{{\left( {{\text{logEC}}_{50} - {\text{X}}} \right) - {\text{Hill }}\;{\text{Slope}}}} }} $$

The assay set up for DAOTA-labeled CarD (CarD-DAOTA) was performed as described above with the following exceptions. The binding curve was performed using 5 nM labeled CarD (Supplemental Fig. S15). The assay was monitored using Perkin Elmer Envision plate reader using a 555 nm excitation filter and 632 nm parallel (S) and perpendicular (P) emission filters. All data was processed as described above.

### Competition experiments

To determine IC_50_ and K_i_ for WT and mutant CarDs, competition experiments were performed as follows. In a 20 µL final volume of 1 × CarD FP Buffer 15 nM MTB RNAP (1.25 × the K_D_) and 300 nM rrnAP3 DNA fragment were kept constant. For determination of K_i_, CarD-BODIPY-FL was tested at 1 nM, 3 nM, and 5 nM final concentrations. WT, R47E, and K90A mutant CarD each were each tested from 20.5 µM down to 625 pM. For determination of small molecule IC_50_ competition experiments, CarD-BODIPY-FL was held constant at 3 nM and DMSO was added to a final concentration of 5% (v/v). For competition experiments with CarD-DAOTA, RNAP was held constant at 40 nM (2 × the observed K_D_; Supplemental Fig. S15). IC_50_ was determined using Eq. (4) and K_i_ was determined from Eq. (5) from 3 independent experiments, each conducted in triplicate, at 3 CarD-BODIPY-FL concentrations which were averaged for the final K_i_. In Eqs (4) and (5), [I]_50_ is the concentration of unlabeled CarD at 50% inhibition (i.e., IC_50_), [L]_50_ is the concentration of free CarD-dye at 50% inhibition, [P]_0_ is the concentration of free CarD-dye at 0% inhibition, and K_D_ is the dissociation constant for the CarD·RPo complex.

4 $$ {\text{Y}} = \frac{100}{{1 + 10^{{\left( {\left( {\log {\text{IC}}_{50} - {\text{X}}} \right) * {\text{Hill}}\;{\text{Slope}}} \right)}} }} $$

5 $$ {\text{K}}_{{\text{i}}} = \frac{{\left[ {\text{I}} \right]_{50} }}{{\frac{{\left[ {\text{L}} \right]_{50} }}{{{\text{K}}_{{\text{D}}} }} + \frac{{\left[ {\text{P}} \right]_{50} }}{{{\text{K}}_{{\text{D}}} + 1}}}} $$

### High throughput screen (HTS)

An initial high throughput screen of the Maybridge 24 K (MB24K) library (23,320 compounds) was conducted using the CarD-BODIPY-FL FP assay. The CarD-BODIPY-FL FP assay was conducted at 25 °C, therefore the buffering conditions were altered so that the pH was consistent with conditions tested above at 37 °C. A shift in K_D_ for the interaction of CarD with the RNAP·DNA complex due to the temperature change (8 nM at 25 °C, Supplemental Fig. S11) resulted in the use of 10 nM of RNAP (1.25 × K_D_) for the screen. CarD-BODIPY-FL was kept constant at 5 nM and rrnAP3 at a constant 300 nM. All other buffer conditions were as described above.

First, 10 µL of 2 × MTB RNAP was added to the 384-well plate using a multidrop dispenser (Thermo Fisher Scientific) followed by 200 nL of compound or DMSO using a Biomek FX HDR pintool instrument (Beckman Coulter) so that final concentration in the assay was 20 µM. Compounds were allowed to incubate with MTB RNAP for 20 min before addition of 10 µL of 2 × CarD-BODIPY-FL and DNA, again with the multidrop dispenser to a final volume of 20.2 µL. For negative controls DMSO without compound was used and for positive controls MTB RNAP was excluded (due to the higher cost and effort to prepare the RNAP). CarD shows essentially no binding to DNA at DNA concentrations up to 30 µM (Fig. 3B). Reactions were allowed to come to equilibrium at 25 °C for 2 h, after which plates were read on a PHERAstar FSX plate reader using 485/20 nm excitation and 520/20 nm emission fluorescence polarization filters. The z-score (Z) was calculated using Eq. (6), where µ_P_ and µ_N_ are the means and σ_P_ and σ_N_ are the standard deviations of the positive and negative controls respectively.

6 $$ {\text{Z}} = 1 - \frac{{3\left( {\sigma_{{\text{P}}} + \sigma_{{\text{N}}} } \right)}}{{\left| {\mu_{{{\text{P}} - }} \mu_{{\text{N}}} } \right|}} $$

The following changes were made when screening with CarD-DAOTA. For primary screening, compounds were added to the plate as described above followed by 10 µL of 2 × CarD-DAOTA and RNAP. Since the dynamic range for the CarD DAOTA assay was significantly smaller than the CarD-BODIPY-FL assay 80 nM RNAP or ~ 2 × K_D,app_ was used. This was allowed to incubate for 20 min followed by the addition of 10 µL 2 × rrnAP3 DNA. For negative controls DMSO without compound was used. DNA was excluded from the positive controls instead of RNAP. CarD exhibits negligible binding to RNAP in the absence of DNA at concentrations below 500 nM (Fig. 3B). The reaction was allowed to incubate for an additional 2 h before reading. For detailed information on confirmation, counter screening, and concentration response screen please refer to the Supplemental Materials and Methods. In brief, compounds which were selected for confirmation were tested in triplicate at a single concentration (20 µM), additionally a counter screen was run where compound was preincubated with probe and the parallel and perpendicular intensities were measured. Compounds which did not interfere with the fluorescent Card were tested at 8 concentrations in duplicate for concentration dependent inhibition.

### In vitro transcription

An in vitro plasmid-based transcription assay using a Malachite Green Aptamer (MGA) gene that was previously developed for high-throughput screening was modified for these studies^20^. The transcription template plasmid (pMGA4-Mt-rrnA3-SynBx3), containing the MTB *rrnA* P3 (− 55 to + 15) ribosomal RNA promoter followed by four consecutive repeats of DNA encoding the MGA and three consecutive repeats of the *synB* artificial terminator, sequence was prepared. Please refer to Supplemental Materials and Methods for a detailed protocol for assay set up.

## Supplementary Information

Supplementary Information.

## References

[1] Chopra I (2007). Bacterial RNA polymerase: a promising target for the discovery of new antimicrobial agents. Curr. Opin. Invest. Drugs.

[2] Ma C, Yang X, Lewis PJ (2016). Bacterial transcription as a target for antibacterial drug development. Microbiol. Mol. Biol. Rev..

[3] World-Health-Organization. *Global tuberculosis Report 2019*. (2019).

[4] Weiss LA (2012). Interaction of CarD with RNA polymerase mediates Mycobacterium tuberculosis viability, rifampin resistance, and pathogenesis. J. Bacteriol..

[5] Hubin EA (2017). Structure and function of the mycobacterial transcription initiation complex with the essential regulator RbpA. Elife.

[6] Davis E, Chen J, Leon K, Darst SA, Campbell EA (2015). Mycobacterial RNA polymerase forms unstable open promoter complexes that are stabilized by CarD. Nucleic Acids Res.

[7] Rammohan J, Ruiz Manzano A, Garner AL, Stallings CL, Galburt EA (2015). CarD stabilizes mycobacterial open complexes via a two-tiered kinetic mechanism. Nucleic Acids Res.

[8] Bae B (2015). CarD uses a minor groove wedge mechanism to stabilize the RNA polymerase open promoter complex. Elife.

[9] Srivastava DB (2013). Structure and function of CarD, an essential mycobacterial transcription factor. Proc Natl Acad Sci U S A.

[10] Zhu D, Garner A, Galburt E, Stallings C (2019). CarD contributes to diverse gene expression outcomes throughout the genome of Mycobacterium tuberculosis. Proc. Natl. Acad. Sci. USA.

[11] Stefan MA, Ugur FS, Garcia GA (2018). Source of the fitness defect in rifamycin-resistant *Mycobacterium tuberculosis* RNA polymerase and the mechanism of compensation by mutations in the beta' Subunit. Antimicrob. Agents Chemother..

[12] Stallings CL (2009). CarD is an essential regulator of rRNA transcription required for Mycobacterium tuberculosis persistence. Cell.

[13] Garner AL, Weiss LA, Manzano AR, Galburt EA, Stallings CL (2014). CarD integrates three functional modules to promote efficient transcription, antibiotic tolerance, and pathogenesis in mycobacteria. Mol. Microbiol..

[14] Gulten G, Sacchettini JC (2013). Structure of the Mtb CarD/RNAP beta-lobes complex reveals the molecular basis of interaction and presents a distinct DNA-binding domain for Mtb CarD. Structure.

[15] Boyaci H (2018). Fidaxomicin jams *Mycobacterium tuberculosis* RNA polymerase motions needed for initiation via RbpA contacts. Elife.

[16] Buurman ET (2012). Novel rapidly diversifiable antimicrobial RNA polymerase switch region inhibitors with confirmed mode of action in *Haemophilus influenzae*. J. Bacteriol..

[17] Lin W (2018). Structural basis of transcription inhibition by Fidaxomicin (Lipiarmycin A3). Mol. Cell.

[18] Jadhav A (2010). Quantitative analyses of aggregation, autofluorescence, and reactivity artifacts in a screen for inhibitors of a thiol protease. J. Med. Chem..

[19] Stallings CL, Glickman MS (2011). CarD: a new RNA polymerase modulator in mycobacteria. Transcription.

[20] Scharf NT, Molodtsov V, Kontos A, Murakami KS, Garcia GA (2017). Novel chemical scaffolds for inhibition of rifamycin-resistant RNA polymerase discovered from high-throughput screening. SLAS Discov..

[21] Desikan P, Rangnekar A (2018). Host-targeted therapy for tuberculosis: Time to revisit the concept. Indian J. Med. Res..

[22] Stefan MA, Ugur FS, Garcia GA (2018). Source of the fitness defect in rifamycin-resistant *Mycobacterium tuberculosis* RNA polymerase and the mechanism of compensation by mutations in the beta' Subuni. Antimicrob. Agents Chemother..

