# Supplementary material for: High-throughput screening to discover inhibitors of the CarD·RNA polymerase protein–protein interaction in Mycobacterium tuberculosis
Source: Sci Rep. 2020 Dec 4;10:21309. doi: 10.1038/s41598-020-78269-3 (PMC7718890; doi:10.1038/s41598-020-78269-3)
Supplement: Supplementary file 1 — Supplementary Information. [file 41598_2020_78269_MOESM1_ESM.docx]

**Supplemental Materials and Methods**

High-throughput Screening to Discover Inhibitors of the CarD•RNA Polymerase Protein-Protein Interaction in *Mycobacterium tuberculosis*

Maxwell A. Stefan, Glory M. Velazquez, and George A. Garcia*

Department of Medicinal Chemistry, University of Michigan, Ann Arbor, MI, USA

*Correspondence to: gagarcia@umich.edu

**Methods**

*RbpA Protein Purification*

RNA polymerase binding protein (RbpA) from *Mycobacterium smegmatis* was purified in a similar fashion to that described by Dey et al.[1] In brief, pETMsRbpA transformed BL21(DE3) cells were grown to an OD_600_ of 0.6 after which cells were induced at 30°C for 4 hours with 1 mM IPTG. Cells from 1 L of culture were resuspended in 20 mL of lysis buffer and disrupted by sonication. Clarified lysate was filtered and applied to a 1 mL HisTrap HP column and eluted with a 10-column volume linear gradient of 20 to 500 mM imidazole in lysis buffer. Fractions with RbpA were concentrated and applied to a Superdex 200 10/300 GL column equilibrated with SEC buffer. Purified RbpA stored at -80°C.

*Confirmation, Counter Screen, and Concentration Response Screen*

Compounds which met hit criteria (**Figure 5** and **Supplemental Figure 14**) were tested as in the primary screen in triplicate. A counter screen was conducted to detect compound interference with CarD-BODIPY-FL fluorescence in the absence of MTB RNAP. This assay was conducted similar to that of the primary and confirmation screen assays. For the confirmation, counter screen, and concentration response testing with CarD-DAOTA, 10 µL 2x CarD-DAOTA and RNAP were added to the plate first followed by compound, as previously described. The plate was allowed to incubate for 20 minutes after which parallel and perpendicular intensities were determined to identify compound which effected fluorescence intensity of the assay. Finally, 10 µL 2x rrnAP3 was added and a final 2-hour incubation step was performed prior to reading as described above.

All compounds which were processed for confirmation were tested in each assay in triplicate. Plates were handled in the same fashion as for the primary screen. Compounds which confirmed and met criteria set for the counter screen were tested in duplicate for concentration-dependent inhibition and were derived from separated stock plates. For concentration-response studies, 200 nL of compound (final concentrations were varied from 150 – 4.2 µM; 1.67x dilutions) or DMSO were dry spotted into 384-well plates prior to addition of any of the assay components. Assay components were then added, incubated and read as described above. The data were fit as described for the CarD titration experiments with the exception that the top and bottom limits were fixed at the average of the negative and positive controls for each curve.

*Reconfirmation*

Reconfirmations with purchased, fresh solid/powder compounds were performed essentially as described above. Fresh compounds were resuspended in DMSO and 1µL deposited into a 384-well plate (final concentration:1000 µM to 0.46 µM). To the 384-well black low volume U-bottom plate 1 µL of compound was added. Using the multidrop, 9 µL of a 22.2 nM RNAP or 80 nM RNAP, for the BODIPY and DAOTA assays respectively, and 11.1 nM CarD-probe solution in 1x CarD FP buffer was added and allowed to incubate with the compounds for 20 minutes at 25°C before the addition of 10 µL of a 2x rrnAP3 DNA solution. The reaction was allowed to come equilibrium over 2 hours at 25°C and then read as previously described.

*In vitro Transcription*

The reaction buffer contained 60 nM pMGA4-Mt-rrnA3-SynBx3, 40 mM Tris-HCl (pH 8.0 at 37°C), 150 mM potassium glutamate, 10 mM MgCl_2_, 0.05% TritonX-100, 25 μg/mL BSA, 1 mM DTT, and 500 µM each NTP. A concentration of 40 nM of MTB RNAP holoenzyme was used and a 3-fold excess of the purified σ factor (SigA) was also added.[2] Compound CCG-249580 (400 µM to 192 nM) was dissolved in DMSO and was incubated with the holoenzyme for 30 minutes before adding the template DNA and NTPs. A final concentration of 4% DMSO was used in all the experiments. Reactions were incubated at 37°C for 90 minutes then halted by incubating on ice for 5 minutes and adding 50 µL of ice-cold malachite green (MG) in water to have a final concentration of 75 µM MG. Fluorescence was measured at excitation and emission wavelengths of 628 nm and 660 nm, respectively, using a BioTek Synergy H1 plate reader. The fluorescence readings were normalized to % activity, plotted against the log [Compound], and fit by nonlinear regression to **Equation (7)** where M0 = log of compound concentration, M1 = log of IC_50_, M2 = hill slope, M3 = lower limit of the curve, and M4 = upper limit of the curve.

**Equation (7)**  $y=M3+\frac{\left( M4-M3 \right)}{\left( {1+10}^{\left( M0-M1 \right)\times M2} \right)}$

In a control experiment, CCG-249580 exhibited no interference with the MG•MGA aptamer binding and fluorescence (data not shown).

*Protein Preparation and NMR analysis*

*E. coli* BL21(DE3) was transformed with the pET19bbps-CarD WT expressing plasmid. Freshly transformed cells were used to inoculate 5 mL of 2xTY + carbenicillin as a pre-culture and were incubated overnight. The next day, a 1 mL inoculum of the 2XTY overnight culture was added to a 100 mL pre-culture of supplemented (vitamins, glucose, trace elements) M9 media, ^15^NH_4_Cl and carbenicillin. This was incubated overnight and the next day 10 mL were used to inoculate the 1 L main culture (same media). At an OD_600_ of 0.6, cells were induced with 1mM IPTG for 4 hours at 28°C. Cells were harvested and CarD was purified following our standard procedure. A final concentration of 150 µM CarD in NMR buffer (100 mM NaCl, 10mM Tris pH 6.5) and 10% D_2_O was used for the ^1^H-^15^N HSQC analyses. Spectra were obtained of CarD alone and CarD in the presence of compound at 4 different concentrations (25 µM-100 µM) with a final DMSO percentage of 3.96%. All NMR spectra were recorded on a Bruker 800 spectrometer equipped with an Ascend magnet with an active shield and a Bruker NEO console. It is equipped with 5mm triple resonance inverse detection TCI cryoprobe with automatic tuning and matching. Standard double-resonance NMR methods were used for data acquisition, processing, and NMR chemical shift assignments. Spectra were processed by using the program MNova.

1 Dey, A., Verma, A. K. & Chatterji, D., Molecular insights into the mechanism of phenotypic tolerance to rifampicin conferred on mycobacterial RNA polymerase by MsRbpA, *Microbiology* **157**, 2056-2071, 2011).

2 Scharf, N. T., Molodtsov, V., Kontos, A., Murakami, K. S. & Garcia, G. A., Novel Chemical Scaffolds for Inhibition of Rifamycin-Resistant RNA Polymerase Discovered from High-Throughput Screening, *SLAS Discov* **22**, 287-297, 2017).

| Name | Sequence | Purpose |
| --- | --- | --- |
| CarD T8C FOR | ATTTTCAAGGTCGGAGACTGCGTTGTCTATCCACACCAC | CarD T8C Mutagenesis |
| CarD T8C REV | GTGGTGTGGATAGACAACGCAGTCTCCGACCTTGAAAAT | CarD T8C Mutagenesis |
| CarD T24C FOR | GTCGAGGCGATCGAATGCCGGACCATCAAAGGGG | CarD T24C Mutagenesis |
| CarD T24C REV | CCCCTTTGATGGTCCGGCATTCGATCGCCTCGAC | CarD T24C Mutagenesis |
| CarD T26C FOR | CGATCGAAACCCGGTGCATCAAAGGGGAAC | CarD T26C Mutagenesis |
| CarD T26C REV | GTTCCCCTTTGATGCACCGGGTTTCGATCG | CarD T26C Mutagenesis |
| CarD D68C FOR | CGGGCAGGAAGGCCTGTGCAAGGTTTTCCAGG | CarD D68C Mutagenesis |
| CarD D68C REV | CCTGGAAAACCTTGCACAGGCCTTCCTGCCCG | CarD D68C Mutagenesis |
| CarD T152C FOR | GACGCCAAAGCCGAGTGCATCCTTGACGAGG | CarD T152C Mutagenesis |
| CarD T152C REV | CCTCGTCAAGGATGCACTCGGCTTTGGCGTC | CarD T152C Mutagenesis |
| CarD S162C FOR | GCCGCCGCGTGTTGAGGATCCGGCTGCTAACAAAGC | CarD S162C Mutagenesis |
| CarD S162C REV | GCTTTGTTAGCAGCCGGATCCTCAACACGCGGCGGC | CarD S162C Mutagenesis |
| rrnAP3 FP TOP | GATGACCGAACCTGGTCTTGACTCCATTGCCGGATTTGTATTAGACTGGCAGGGTTGCCCC | FP Assay DNA |
| rrnAP3 FP BOTTOM | GGGGCAACCCTGCCAGTCTAATACAAATCCGGCAATGGAGTCAAGACCAGGTTCGGTCATC | FP Assay DNA |
| Artificial Bubble TOP | GGCTCTTGACAAAAGTGTTAAATTGTGCT**ATACTGGGATGG**TATGGATGACAGAATTCGG | FP Assay DNA |
| Artificial Bubble BOTTOM | CCGAATTCTGTCATCCATAGGTAGGGTCATAAGCACAATTTAACACTTTTGTCAAGAGCC | FP Assay DNA |

**Supplemental Table 1: Primers used. Mutagenesis sites are underlined.**

| CarD Variant | m/z | | Percent Labeling | |
| --- | --- | --- | --- | --- |
|  | **Calculated** | **Experimental** | **LCMS** | **UV/Vis** |
| WT | 18198.68 | 18198.95 | NA | NA |
| R47E | 18171.60 | 18171.93 | NA | NA |
| K90A | 18141.58 | 18141.70 | NA | NA |
| D68C | 18186.73 | ND | NA | NA |
| T8C-BODIPY FL | 18489.81 | 18489.90 | 91% | 96% |
| T24C-BODIPY-FL | 18489.81 | 18490.08 | 97% | 90% |
| T26C-BODIPY-FL | 18489.81 | 18490.08 | 98% | 67% |
| D68C-BODIPY-FL | 18475.83 | 18475.86 | 100% | 88% |
| T152C-BODIPY-FL | 18489.81 | 18489.91 | 98% | 87% |
| S162C-BODIPY-FL | 18503.85 | 18504.08 | 52% | 37% |
| D68C-DAOTA | 18613.84 | 18613.94 | 100% |  |

**Supplemental Table 2: Characterization of mutant and labeled CarD variants.**

Mass of CarD variants derived from theoretical values, empirical masses from LCMS and % labeling from LCMS and UV spectroscopy.

| **CarD-BODIPY-FL** | | | |
| --- | --- | --- | --- |
| Name | **Cluster** | **CCG Sample IC_50_ (µM)** | **Powder Sample IC_50_ (µM)** |
| CCG-52160 | I | 37 | 680 |
| CCG-52573 | I | 78 | 160 |
| CCG-251660 | II | 106 | 37 |
| CCG-53654 | III | 15 | 188 |
| CCG-247600 | IV | 250 | 132 |
| CCG-239496 | V | 90 | 47 |
| CCG-52363 | VI | 60 | 200 |
| CCG-42210 | VII | 106 | 85 |

**Supplemental Table 3: Eight initial hits from the CarD-BODIPY-FL screen of the MB24K.**

| Name | Structure | MW (g/mol) | SFI | cLogD (7.4) | Ar Rings | H-bond acceptors | H-bond donors | tPSA |
| --- | --- | --- | --- | --- | --- | --- | --- | --- |
| CCG-237488 |  | 278.3 | 5.43 | 2.43 | 3 | 3 | 2 | 66.91 |
| CCG-249580 | 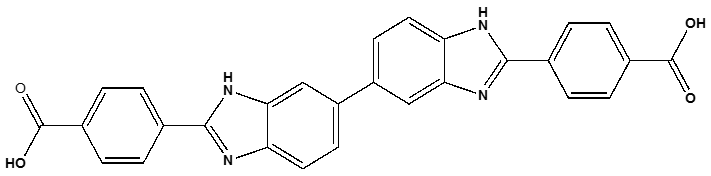 | 474.5 | 4.74 | -1.26 | 6 | 6 | 4 | 131.96 |
| 019-738-824 | 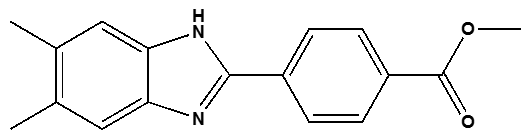 | 280.3 | 7.31 | 4.31 | 3 | 3 | 1 | 54.98 |
| 008-371-831 | 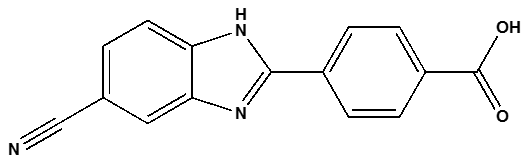 | 263.1 | 2.61 | -0.39 | 3 | 4 | 2 | 89.77 |
| 046-418-303 | 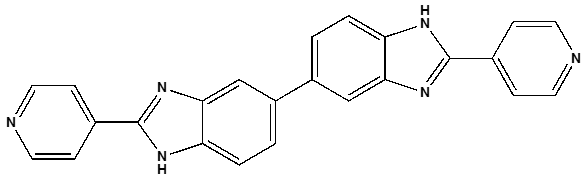 | 388.1 | 9.80 | 3.8 | 6 | 4 | 2 | 83.14 |

**Supplemental Table 4: Physiochemical properties of reconfirmed hits from CarD-DAOTA screen and three CCG-249580 analogues.**

Physicochemical properties (cLogD_7.4_, SFI, tPSA) were calculated using the Marvin Suite from Chemaxon.

**A**

**B**

**C**

**D**

**WT CarD**

**Supplemental Figure 1: LCMS Characterization of WT CarD.**

**A)** LC trace, **B)** fragmentation by MS, **C)** deconvoluted protein m/z, and **D)** magnification of peaks proximal to the protein m/z.

**A**

**B**

**C**

**D**

**CarD R47E**

**Supplemental Figure 2: LCMS Characterization of CarD R47E.**

**A)** LC trace, **B)** fragmentation by MS, **C)** deconvoluted protein m/z, and **D)** magnification of peaks proximal to the protein m/z.

**A**

**B**

**C**

**D**

**CarD K90A**

**Supplemental Figure 3: LCMS Characterization of CarD K90A.**

**A)** LC trace, **B)** fragmentation by MS, **C)** deconvoluted protein m/z, and **D)** magnification of peaks proximal to the protein m/z.

**A**

**B**

**C**

**D**

**CarD T8C BODIPY**

**CarD T8C***

*****

**Supplemental Figure 4: LCMS Characterization of CarD T8C BODIPY.**

**A)** LC trace, **B)** fragmentation by MS, **C)** deconvoluted protein m/z, and **D)** magnification of peaks proximal to the protein m/z.

**A**

**B**

**C**

**D**

**CarD T24C BODIPY**

**CarD T24C***

*****

**Supplemental Figure 5: LCMS Characterization of CarD T24C BODIPY.**

**A)** LC trace, **B)** fragmentation by MS, **C)** deconvoluted protein m/z, and **D)** magnification of peaks proximal to the protein m/z.

**A**

**B**

**C**

**D**

**CarD T26C BODIPY**

**CarD T26C***

*****

**Supplemental Figure 6: LCMS Characterization of CarD T26C BODIPY.**

**A)** LC trace, **B)** fragmentation by MS, **C)** deconvoluted protein m/z, and **D)** magnification of peaks proximal to the protein m/z.

**A**

**B**

**C**

**D**

**CarD D68C BODIPY**

**Supplemental Figure 7: LCMS Characterization of CarD D68C BODIPY.**

**A)** LC trace, **B)** fragmentation by MS, **C)** deconvoluted protein m/z, and **D)** magnification of peaks proximal to the protein m/z.

**A**

**B**

**C**

**D**

**CarD T152C BODIPY**

**CarD T152C***

*****

**Supplemental Figure 8: LCMS Characterization of CarD T152C BODIPY.**

**A)** LC trace, **B)** fragmentation by MS, **C)** deconvoluted protein m/z, and **D)** magnification of peaks proximal to the protein m/z.

**A**

**B**

**C**

**D**

**CarD S162C BODIPY**

**CarD S162C**

**Supplemental Figure 9: LCMS Characterization of CarD S162C BODIPY.**

**A)** LC trace, **B)** fragmentation by MS, **C)** deconvoluted protein m/z, and **D)** magnification of peaks proximal to the protein m/z.


**A**

**B**

**C**

**D**

**CarD D68C DAOTA**

**Supplemental Figure 10: LCMS Characterization of CarD D68C DAOTA.**

**A)** LC trace, **B)** fragmentation by MS, **C)** deconvoluted protein m/z, and **D)** magnification of peaks proximal to the protein m/z.


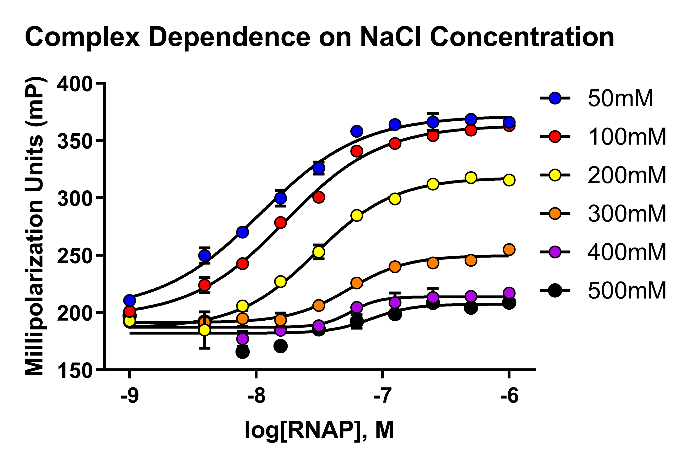
**A**


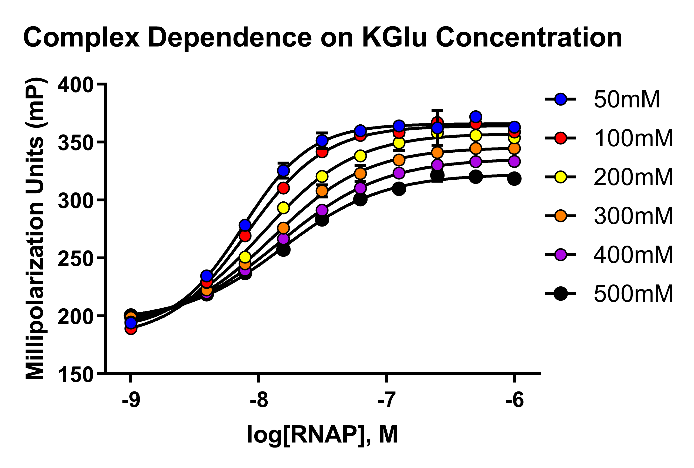
**B**


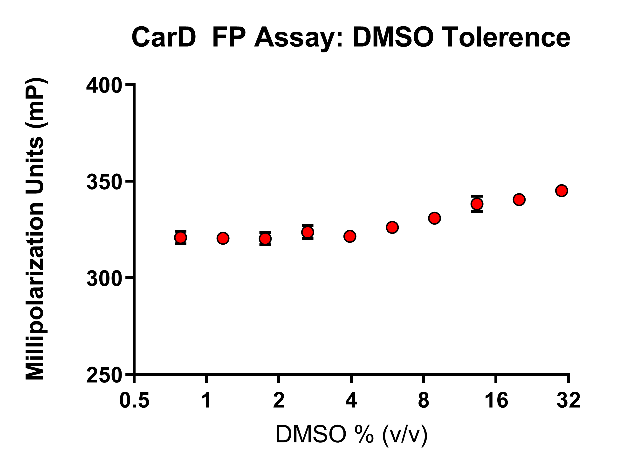


**C**

**Supplemental Figure 10: Optimization of the CarD-BODIPY-FL FP assay.**

**A)** Stability of the CarD-BODIPY-FL FP assay with NaCl titration. **B)** Stability of CarD-BODIPY-FL FP assay with KGlu titration. **C)** Tolerance of CarD-BODIPY-FL assay to the presence of increasing concentrations of DMSO.


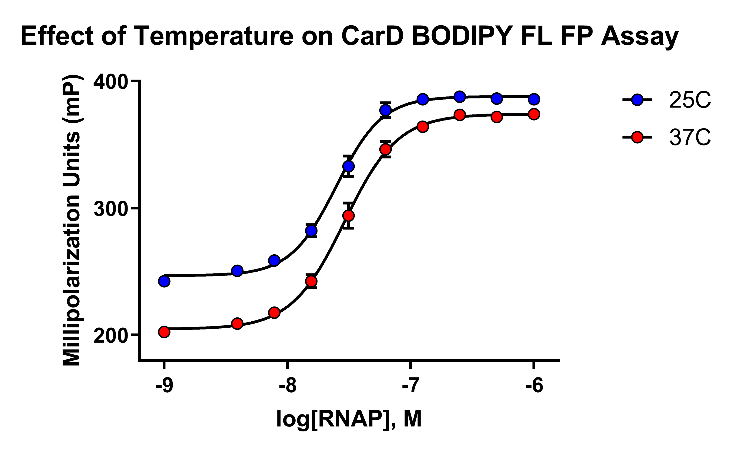
**A**


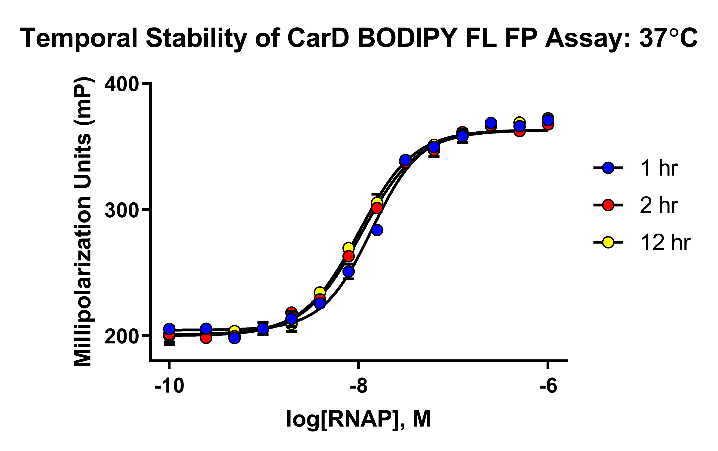
**B**


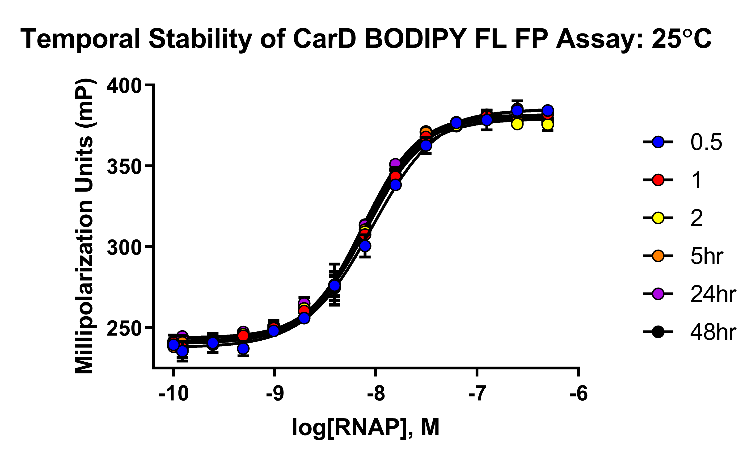
**C**

**Supplemental Figure 11: Temperature and Temporal Stabilities of the CarD-BODIPY-FL FP Assay.**

**A)** Effect of temperature on CarD-BODIPY-FL FP Assay. **B)** Temporal stability of the CarD-BODIPY-FL FP Assay at 37°C over 12 hrs. **C)** CarD-BODIPY-FL FP assay temporal stability over 48 hours.


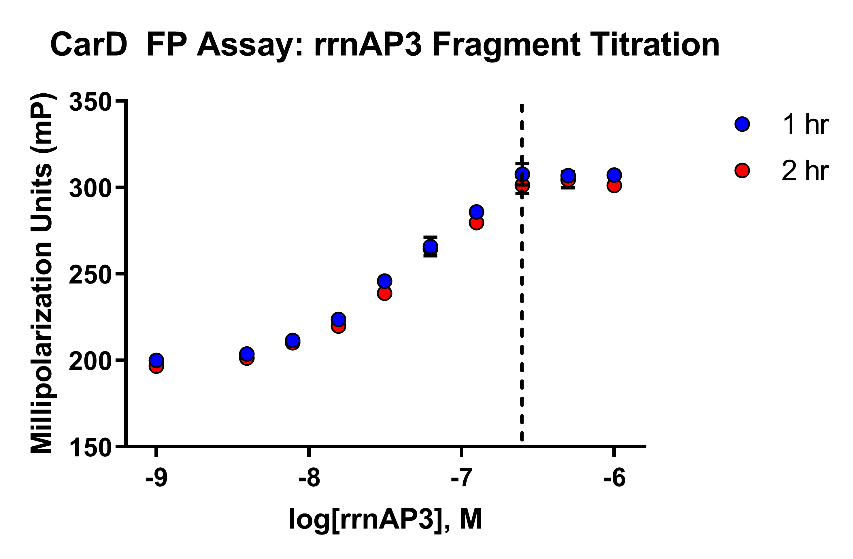


**Supplemental Figure 12: Titration of rrnAP3 DNA in CarD FP assay.**

RNAP is present at 15 nM and CarD-BODIPY-FL at 3 nM. The dashed line is at 250 nM and represents the threshold for the minimum amount of rrnAP3 DNA which needs to be present to maintain signal stability.

**Supplemental Figure 13: Detailed schematic of criteria for selection of hits from the CarD-BODIPY-FL primary screen of the MB24K library to compound ordering (compounds removed with filter(s)).**


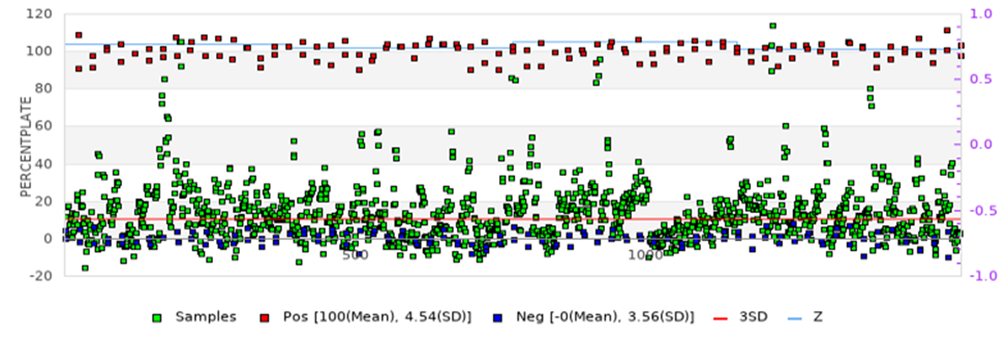
**A**


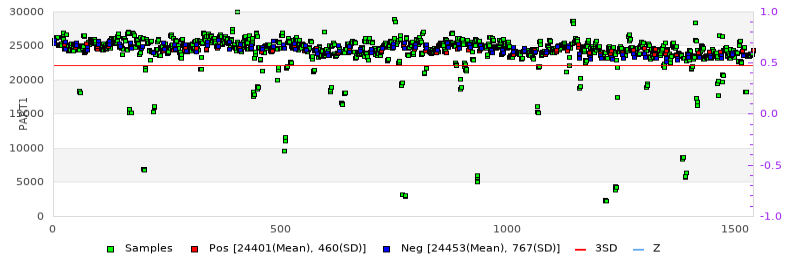
**B**


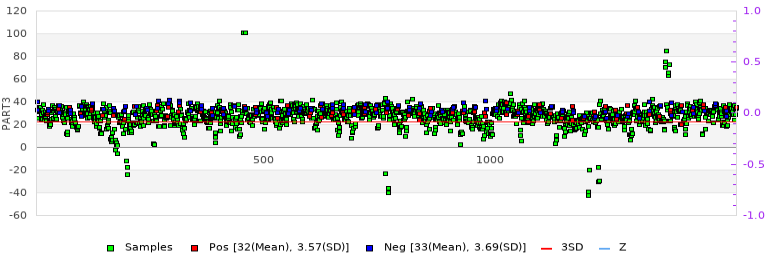
**C**

**Supplemental Figure 14: Retest plate data for confirmation from the CarD-BODIPY-FL primary screen of the MB24K library.**

**A)** All compounds which were tested for confirmation in triplicate. **A)** All compounds which were screened in triplicate. **B & C)** Effect of compounds tested against CarD-BODIPY-FL alone in the counter screen: **B)** parallel fluorescence intensity, **C)** polarization **(**mP).

**A**

** B**

**C**

**Supplemental Figure 15: Optimization of the CarD-DAOTA FP assay.**

**A)** Stability of the CarD-BODIPY-FL FP assay with NaCl titration. **B)** Stability of CarD-BODIPY-FL FP assay with KGlu titration. **C)** Tolerance of CarD-BODIPY-FL assay to the presence of increasing concentrations of DMSO.

**Supplemental Figure 16: Detailed schematic of criteria for selection of hits from the CarD-DAOTA screen of 852 hits that interfered with the BODIPY-FL fluorescence.**


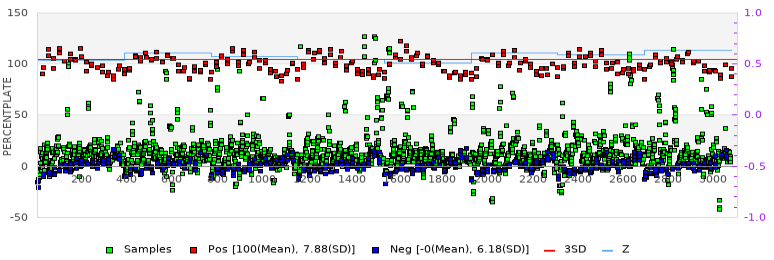
**A**

**
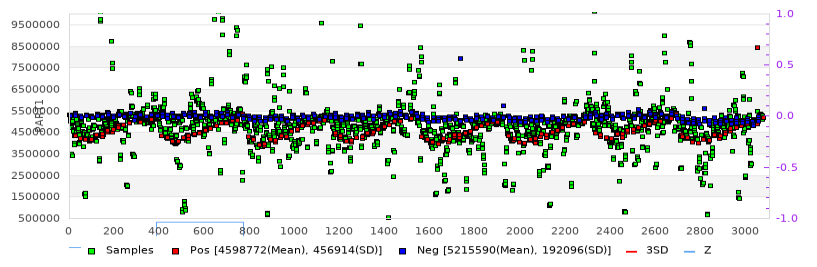
B**


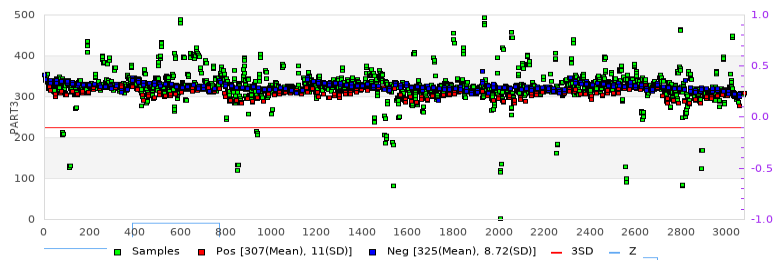
**C**

**Supplemental Figure 17: Plate data for the CarD-DAOTA screen of 852 hits that interfered with the BODIPY-FL fluorescence.**

**A)** All compounds which were screened in triplicate. **B & C)** Effect of compounds tested against CarD-DAOTA alone in the counter screen: **B)** parallel fluorescence intensity, **C)** polarization **(**mP).

(We note a systematic variation in the positive and negative controls that is repeated on each plate. The source of this variation is unclear; however, the magnitude of the variation is small and the overall Z-score for each plate is > 0.5 indicating that the screen is robust.)


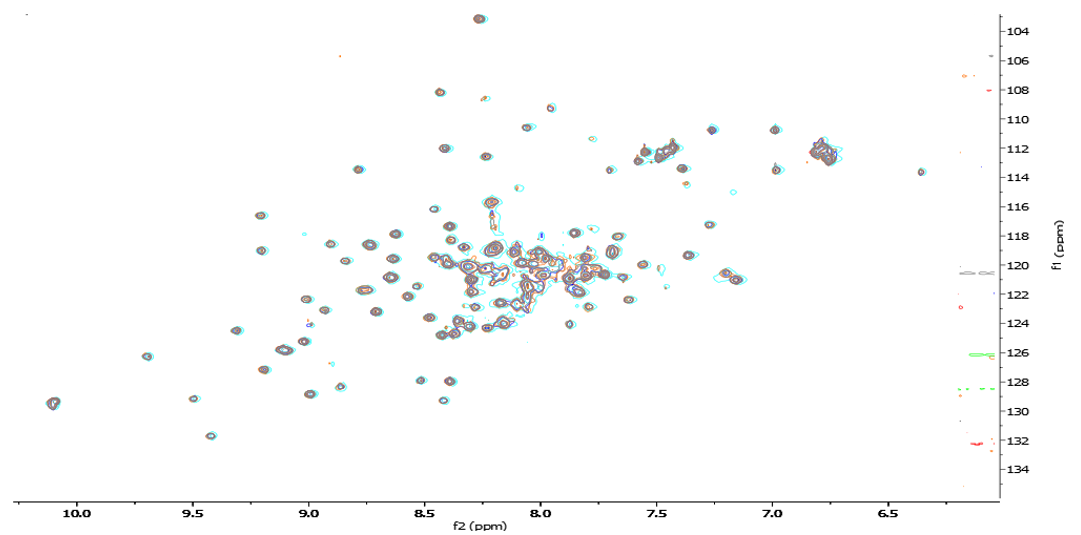


**Supplemental Figure 18: Overlay of the CarD ^1^H-^15^N HMQC spectra obtained from the CCG-249580 titration experiments.**

No ^1^H and ^15^N chemical shift changes were observed upon titrating CCG-249580 into an ^15^N-labeled CarD solution. The data were evaluated by overlaying 5 spectra obtained after testing CCG-249580 at 4 different concentrations (25 µM- 100 µM) and CarD alone.
